# Influence of Organized vs Non Organized Physical Activity on School Adaptation Behavior

**DOI:** 10.3389/fpsyg.2020.550952

**Published:** 2020-11-19

**Authors:** Adrian A. Mosoi, Jürgen Beckmann, Arash Mirifar, Guillaume Martinent, Lorand Balint

**Affiliations:** ^1^Department of Psychology, Education and Teacher Training, Faculty of Psychology and Education Sciences, Transilvania University of Braşov, Braşov, Romania; ^2^Department of Sport and Health Sciences, Chair of Sport Psychology, Technical University of Munich, Munich, Germany; ^3^Faculty of Health and Behavioural Sciences, School of Human Movement and Nutrition Sciences, University of Queensland, Brisbane, QLD, Australia; ^4^Department of Physical Education and Sport Sciences (PESS), University of Limerick, Limerick, Ireland; ^5^Laboratory of Vulnerabilities and Innovation in Sport, University of Claude Bernard Lyon 1 – University of Lyon, Lyon, France; ^6^Department of Physical Education and Special Motricity, Faculty of Physical Education and Mountain Sports, Transilvania University of Braşov, Braşov, Romania

**Keywords:** physical activity, disabilities, conduct disorders, school behavior, adolescents

## Abstract

It is now well-established that physical activity has positive effects on both physical and mental health. However, the influence of organized physical activity (i.e., programs controlled and supervised by a trainer) on school adaptive behavior of adolescents with disabilities and/or behavioral disorders remains unclear. School behavior adaptation involves the ability to learn, conform to school norms and manage school activities without major behavior conflicts. A cross-sectional study was conducted to test the differences between organized physical activity and non-organized physical activity in an after school program. Eighty Romanian adolescents were recruited and allocated to three groups: (a) with disabilities [Ds; *N* = 17, *M*_age_ = 14.55 years (*SD* = 1.16), 12 males and 5 females], (b) with conduct disorders [CDs, *N* = 21, *M*_age_ = 14.52 years (*SD* = 1.11) 16 males and 5 females], and (c) participants who had not shown signs of conduct disorders or disabilities [as a control group; *N* = 42, *M*_age_ = 14.2 years (*SD* = 0.46) 20 males and 22 females]. Personality traits, school behavior, and sensorimotor coordination were assessed by using the Eysenck personality questionnaire—junior scale, school in-adaptability questionnaire scale, and Vienna Test System Sport (SMK—sensorimotor coordination test) respectively. Multivariate analysis of variance MANOVA (3 × 3) and discriminant analysis were used to examine differences between the psychological and sensorimotor coordination outcomes across three groups and three types of physical activity context: (a) organized physical activity, (b) non-organized physical activity, and (c) no physical activity. The findings indicate that not participating in an organized physical activity program results in a reduced level of physical mobility and consequently is associated with maladaptive social and psychological outcomes. Thus, we argued that attending in an organized physical activity program is more beneficial for participants with disabilities and/or behavior disorders, due to an increase in the probability of school integration and development of their motor skills. Clearly more research is needed in order to investigate these effects in neurophysiological levels.

## Introduction

Adolescence has often been described as a period of turmoil during which: (a) young people are more at risk for emotional maladjustment, and (b) physical activity typically declines or is completely avoided (Skinner and Piek, 2001). Particularly among adolescents with either disabilities or conduct disorders, the aforementioned issues could be more prominent. Thus, it is assumed that being involved in an organized practice of physical activity during adolescence could prevent such maladjustments (Moore and Werch, 2005; Taliaferro et al., 2008). Participating in organized physical activity during adolescence at schools in after-school programs (in addition to the regular physical activity sessions included in the compulsory teaching program) has been shown to promote the development of psychological skills (Owen et al., 2014; Esteban-Cornejo et al., 2015; Hills et al., 2015; Timo et al., 2016), and improve school behavior (Rasberry et al., 2011; Beckmann and Elbe, 2015; Naylor et al., 2015), as well as motor function (Hands et al., 2009; McIntyre et al., 2015). In contrast, lack of physical activity (inactivity) has been associated with mental health risks (Delisle et al., 2010; Biddle and Asare, 2011; Elinder et al., 2011), and impairment of learning (Davis et al., 2011). Physical activities in an organized setting refer to the use of sports halls or fields with availability of appropriate equipment’s for sports activities (team or individual) and physical activity lessons. In these types of activities, under the supervision of a trainer, facilities are adapted to meet a participant’s demands. Participants, in this organized setting, usually are required to follow their trainer’s guidance (Vella et al., 2016) to perform extracurricular activities (Rasberry et al., 2011) and the environment is usually competitive (Eime et al., 2013). However, this is not the case in non-organized activities, whereas the type of activities are indicated by lack of practical instructor training or lack of structured activities (Walters et al., 2009).

Although, the benefits of physical activity in school adjustment and academic performance are well documented for typically developing adolescents (Fox et al., 2010; Rasberry et al., 2011; Booth et al., 2014; Jewett et al., 2014), the effects of physical activity on adolescents with conduct disorders and adolescents with disabilities are less understood (Stathopoulou et al., 2006; Kasser and Lytle, 2013; Clow and Edmunds, 2014). By considering that the adolescents with the above-mentioned conditions are part of the regular school system, there is high demand for research in this area (Malone et al., 2012; Sachlin and Lexell, 2015).

The present study focuses on school adjustment comparing adolescent groups in terms of the variables included in the study such as personality traits: extraversion; neuroticism; and psychoticism (Rhodes and Smith, 2006), school behavior: rebelliousness and school neuroticism (Clinciu, 2014), and motor functions: sensorimotor coordination (Perkins and Noam, 2007). These variables were reported (Rhodes and Smith, 2006; Reiss, 2009; Poitras et al., 2016) by distinct groups of adolescents (i.e., adolescents with disabilities, adolescents with conduct disorders, typically developing adolescents).

### Physical Activity Involvement Among Adolescents With Disabilities

In general, it has been shown that adolescents with physical disabilities are less involved in physical activities, particularly in school. For instance, McIntyre et al. (2015) reported that adolescents with decreased motor abilities tend to avoid participating in physical activities. The rate of participation in leisure activities of adolescents with disabilities is associated with several variables, such as the degree of coordination (e.g., sensorimotor coordination), communication capacity, cognitive functions, and poor rehabilitation outcomes (Henderson and Bryan, 1997). These variables are particularly important for social integration of adolescents with disabilities (Bult et al., 2011) given the fact that the level of social inclusion is dependent on community access rather than on the high number of friendships (Simplican et al., 2015).

Numerous studies have provided evidence that participation in leisure physical activities increases well-being and life satisfaction of adolescents with disabilities (Johnson, 2009; Yazicioglu et al., 2012). For example, Dahan-Oliel and Shikako-Thomas (2012) highlighted significant improvements not only in motor functions of adolescents with disabilities, but also regarding their perceived quality of life and friendships as a consequence of participating in an organized physical activity program during the school programs. Additionally, other researchers (e.g., Shapiro and Martin, 2014) have reported participation in physical activity fosters growth and development of friendships outside of these settings. Furthermore, Edwards (2015) pointed out that physical activity could allow consolidation of the abilities required for social co-existence and for increasing social cohesion.

### Physical Activity Involvement Among Adolescents With Conduct Disorders

One disability that affects around 5% of adolescents worldwide is conduct disorder (Erskine et al., 2017). Conduct disorder is defined as a repetitive and persistent pattern of behavior in which the basic rights of others or major appropriate norms are violated, such as failure to conform to norms, or cruel and aggressive behavior (American Psychiatric Association [APA], 2013). Research has shown that conduct disorder can occur in childhood and adolescence, and adolescents with learning disabilities have an increased risk of developing associated conduct disorders (Sadock and Sadock, 2007; American Psychiatric Association [APA], 2013). According to Mahoney and Stattin (2000), participation in organized leisure activities (e.g., sports) was negatively associated with antisocial behavior compared with participation in unorganized leisure activities (e.g., recreation centers). However, the efficacy of social rehabilitation and reintegration through physical activity remains a challenge to develop sufficient empirical support, especially for disadvantaged groups (Archie et al., 2007; Desha et al., 2007). The shared nature of benefits in school sport participation has been demonstrated in several other studies. Alternatively, Jewell et al. (2015) highlighted that school sport participation during adolescence was significantly associated with higher self-rated mental health in young adulthood. For instance, Samek et al. (2015) found that adolescents involved in sports showed fewer conduct disorder symptoms than those not involved in sports. Furthermore, it was found that conflict behavior rates peak when adolescents with conduct disorders participate in unsupervised after school programs (Jewell et al., 2015). Therefore, this means that a careful management of physical activity or sports in an organized framework would be most beneficial for adolescents with conduct disorders improving their opportunities to adapt to school and social norms.

### The Present Study

There are a number of potential reasons for analyzing the benefits of organized physical activity participation in adolescence regarding school integration (Marcus et al., 2000; Reiss, 2009; Biddle and Asare, 2011). To the best of our knowledge no previous studies have yet focused on the relationship between organized physical activity and personality traits, school behavior and sensorimotor coordination among different groups of adolescents (adolescents with disabilities, adolescents with conduct disorders, and adolescents who develop normally). Adolescents who develop normally have no physical or mental disabilities and are free from any diagnosed disorders, are also known as typically developing adolescents (Pan et al., 2015). Moreover, the research conducted on adolescents with disabilities and adolescents with conduct disorders regarding school integration are rather scarce. When considering the fact that there are no differences in the education system between those with and without disabilities, we therefore assumed no differentiation would be necessary between groups. In this study, all adolescents with disabilities or conduct disorders were diagnosed by trained physicians in public hospitals and were assessed by qualified school district personnel according to the guidelines of the Diagnostic and Statistical Manual of Mental Disorders, 5th edition (DSM-V; American Psychiatric Association [APA], 2013). Additionally, school behavior outcomes should be an important factor in the development of the adolescent regarding school integration, acknowledging whether the individual suffers from a disability or a disorder. Therefore, the present research aimed to fill this gap by evaluating the effectiveness of organized physical activity on school behavior, psychological, and sensorimotor coordination among different groups of adolescents: adolescents with physical disabilities (Ds), adolescents with conduct disorders (CDs), and participants who had not shown signs of conduct disorders or disabilities (as a control group; Cs). On the basis of the literature (Heaven et al., 2008; Johnson, 2009; Rockhill et al., 2009; Dahan-Oliel and Shikako-Thomas, 2012; Yazicioglu et al., 2012; Hoolis et al., 2014; Shapiro and Martin, 2014; Edwards, 2015) we hypothesized that: (a) the participants in Ds group will show significantly lower scores of sensorimotor coordination in comparison to other groups, and (b) the participants in Cs group will show higher levels of school behavior in comparison to other groups. We also hypothesized that adolescents participating in organized physical activity will report significantly lower scores of psychoticism, school neuroticism and rebelliousness in comparison to adolescents not taking part in physical activity.

## Materials and Methods

### Participants

Originally 120 Romanian adolescents voluntarily participated, 80 of which completed all questionnaires and were therefore included in the present study (48 boys and 32 girls; *M*_age_ = 14.3 years, *SD* = 0.9): (a) Seventeen adolescents with physical disabilities (Ds) diagnosed with associated motor deficiencies. (b) Twenty-one adolescents with conduct disorders (CDs). (c) Forty-two participants who had not shown signs of conduct disorders or disabilities (as a control group; Cs). With approval and financial support from both the European Social Fund and the Romanian Government, a group of adolescents participated in our research study investigating the relevance of the qualitative level of psychological and motor components designed to improve school adaptation in this age group. Therefore, only adolescents with disabilities and conduct disorders, as well as typically developing adolescents were included in the study.

The participants of these three groups were distributed according to the involvement in physical activity after school programs, such as: (a) organized practice of physical activity (OPA)—their performance was controlled by the coach or instructor (controlled physical activity 3–4 times per week); (b) non-organized practice of physical activity (NOPA)—their performance was not controlled by the coach or an adult (unstructured physical activity 1–2 times per week); or (c) no physical activity (NPA)—they were not involved in any physical activity or do not participate in any form of organized sport or physical activity after the school program.

### Instruments

To assess personality traits we used the Romanian validated version of Grigoriu-Şerbănescu (1984) the Junior Eysenck Personality Questionnaire—Junior EPQ (Eysenck and Eysenck, 1975), measuring extraversion (action orientated; enthusiastic; talkative; assertive), neuroticism (irritable, nervous, apprehensive), and psychoticism (hostile, aggressive, impersonal, foolhardy), which was developed for individuals ages 10–18 years old (81 items). Participants responded on a dichotomous scale (yes = 1, no = 0). Cronbach’s alpha was used to determine the internal consistency reliability of the total score. were α = 0.28, α = 0.79, and α = 0.67 for extraversion, neuroticism and psychoticism, respectively. However, due the poor reliability, extraversion was not included in the data analysis. To further assess the internal reliability of the Junior EPQ scores, item analyses were conducted (DeVellis, 2003). To test each item, the following criteria were adopted: (a) a minimum item-total correlation coefficient of *r* = 0.40 and (b) a mean inter-item correlation value of 0.20–0.70 (DeVellis, 2003; Piedmont, 2014). With the scale, the overall value of the corrected item-total correlation was observed to be in the range of 0.23–0.50 for psychoticism and 0.27–0.51 for neuroticism, respectively. After exclusion extraversion, all items of the psychoticism and neuroticism fulfilled these criteria, providing evidence for the reliability of these two scales.

The SIQ—School In-adaptability Questionnaire (Clinciu, 2014) is a Romanian questionnaire consisting of 67 items covering the dimensions of school neuroticism (43 items; e.g., scholar stress, failing, α = 0.90) and rebelliousness (24 items; e.g., school indiscipline, antisocial behavior, α = 0.80). Participants responded on a dichotomous scale (yes = 1, no = 0). Reliability was good in the present study with Cronbach’s α = 0.83 for school neuroticism and α = 0.75 for rebelliousness, respectively. The value of the corrected item-total correlation with the scale overall was observed to be in the range of 0.23–0.60 for school neuroticism and 0.22–0.55 for rebelliousness, respectively. Moreover, all items of the SIQ fulfilled the aforementioned criteria (DeVellis, 2003; Piedmont, 2014), providing evidence for the reliability of these scales.

To assess sensorimotor coordination, we used SMK (short form S1), from Vienna Test Systems Sport of the research Institute of Transilvania University in Braşov. This test is a computerized task using a joystick and was applied to measure the motor control ability (Mosoi and Balint, 2015) by utilizing the feedback in real time of sensorimotor information from the movement currently being executed by the adolescents. With this test, the task was to use a synchronous joystick to maneuver a circular yellow segment that is drawn as a piece of pizza into a required position with a tip touching a bar upright in the upside “T” position. The circle segment, which is standing on its tip, has three types of movement, including tilting to left/right, horizontal movement to left/right and movement along the depth perspective with corresponding change in size randomly made in a three-dimensional space. All the participants were properly instructed on how to use the joysticks. Overall, the test takes 15 min, including 5 min of a practice phase and 10 min of evaluation. A total test score was obtained based on the calculation of the actual positioning time of the segment at the desired position (Schuhfried, 2013).

### Procedure

The current study was approved by the Transilvania University of Braşov (POSDRU/159/1.5/S/134378). The school inspectorate of Braşov County and the principals of the participating adolescents’ schools were approached by a research team at the beginning of the procedure. Participation of adolescents was voluntary. Written informed consent from adolescents and their parents was obtained prior to participation in the study. The inclusion and exclusion criteria of the study were as follows: (a) are in the first year of grammar school; (b) take part in school classes every day; (c) take part in school physical exercise lessons every week; (d) do not take any medication; (e) complete both sessions. The measurements were conducted in 2 consecutive weeks. During the first week, participants completed the psychological questionnaires and during the second week they participated in the sensorimotor coordination assessment. The clinical groups completed the questionnaires on 3 consecutive days. Due to reduced concentration skills, this group required more time for the test than the other groups. Additionally, the sensorimotor test was conducted in individual sessions which took place in a quiet room at school, while questionnaires measurements were taken in group sessions. The teachers, caregivers, and the parents were comprehensively informed about the research project and the conditions of the participation. During the assessments with principals’ agreements, the adolescents completed the EPQ, SIQ, SMK, as well as the demographic questionnaire.

### Sample

The participants were divided into three distinct groups. The first non-clinical group consisted of participants from conventional schools, which were defined as typically developing adolescents. The second group included adolescents with disabilities represented by the “Inclusive School.” These two groups were divided according to the participation on physical activities in after school programs. Lastly, the third group consisted of adolescents with conduct disorder selected from “Adolescents Psychiatry Hospital.” In the first two groups the differences were between specific participation in physical activities in after school programs, where typically developing adolescents were involved in different sports and adolescents with disabilities were involved in exercise therapy to improve especially their mobility. The third group, controlled in a hospital institution, included adolescents with conduct disorders that were involved only in unorganized physical activities, because after school programs are not offered. In Romanian schools, adolescents with disabilities and conduct disorders were included in the compulsory teaching programs. Those with disabilities were taking part in exercise therapy to increase their motor adaptability toward different tasks or to prevent injury risks. Adolescents with conduct disorder, on the other hand, were practicing physical exercise to prevent antisocial interaction (Romanian Law Education, 1/2011, art. 48–50). Both groups participated in the school’s general physical education program 100% of the time. In order to be included in data analysis, participants needed to have met the five criteria, as well as completed both the individual and group sessions.

### Data Analysis

A multivariate analysis of variance (MANOVA) was conducted to examine the main effects of adolescent groups (Ds, CDs, and control group) and physical activity groups (OPA, NOPA, NPA) on all the dependent variables (EPQ, SIQ, and SMK scores). With such a 3 × 3 MANOVA, we also explored the interaction effect of adolescent groups × physical activity groups. In the analyses, in case of significant multivariate effect (*p* < 0.05) *post-hoc* comparisons (Tukey’s HSD) were conducted. Partial eta squared (η*^2^*) provided an index of effect size (Ferguson, 2009). Finally, the MANOVA was complemented by discriminant function analyses (Field, 2013).

## Results

### Descriptive Results

Information, regarding age, sex, parental educational background (primary school; grammar school; university), family structure (mono parental; intact family; divorce) and their current participation status in physical activity (no physical activities; non-physical activity; organized physical activity) was collected before starting the measurements. The Ds, CDs, and Cs were well matched on age and sex, but not on the family structure and parental educational backgrounds. Participants indicated the weekly frequency of organized physical activity (controlled physical activity 3–4 times per week) and non-organized physical activity after school programs (unstructured physical activity 1–2 times per week; not participate in any form of physical activity). The details of the participants included in this study are presented in Table 1.

**TABLE 1:** Descriptive statistics of the participants.

**Group type**		**Ds (*N* = 17) *M* (*SD*)**	**CDs (*N* = 21) *M* (*SD*)**	**Cs (*N* = 42) *M* (*SD*)**	**Overall (*N* = 80)**
Age (year)	Mean (SD)	14.55 (1.16)	14.52 (1.11)	14.2 (0.46)	14.36 (0.90)
Gender	Male	12	16	20	48
	Female	5	5	22	32
Family structure	Mono parental	3	3	13	19
	Intact family	12	7	31	50
	Divorce	–	11	–	11
Parental educational background	Primary school	4	6	4	14
	Grammar school	11	12	20	33
	University	2	3	18	23
No physical activities	Not participate in any form of physical activity	5	–	14	19
Non-organized physical activities	Unstructured physical activity 1–2 times per week	7	21	7	35
Organized physical activities	Controlled physical activity 3–4 times per week	5	–	21	26

**Ds, Disabilities; CDs, Conduct disorder; Cs, control group.**

### Results of Comparison Groups

The results of the Box’s M test (*p* = 0.168) were not significant and provided evidence for the assumption of equality of covariance matrices. Also, the tests of homogeneity of variance for all dependent variables were not significant (psychoticism, *p* = 0.244; neuroticism, *p* = 0.066; school neuroticism, *p* = 0.084; rebelliousness, *p* = 0.653; and sensorimotor coordination, *p* = 0.094). However, results of the MANOVA showed significant main effects for adolescents’ groups [Wilk’s Lambda, Λ = 0.57 *F*(10, 138) = 4.44, *p* < 0.001, partial η*^2^* = 0.24] and physical activity groups [Λ = 0.73, *F*(10, 138) = 2.38, *p* < 0.012, partial η*^2^* = 0.15] as well as a significant effect for the interaction of adolescents’ groups × physical activity groups [Λ = 0.76 *F*(10, 138) = 2.06, *p* < 0.032, partial η*^2^* = 0.13]. Results of *post-hoc* comparisons, using Tukey’s HSD, are presented in Table 2. Results of *post-hoc* comparisons adolescents’ groups showed that: (a) CDs reported significantly higher scores of psychoticism, neuroticism, school neuroticism and rebelliousness than Cs; (b) CDs reported significantly higher scores of school neuroticism than Ds; (c) Cs reported significantly higher scores of sensorimotor coordination than Ds and CDs. The effect size found on adolescents groups was small, η^2^ = 0.18 for rebelliousness (Cs < CDs) and η^2^ = 0.30 for sensorimotor coordination (Ds; CDs < Cs).

**TABLE 2:** The main effects of adolescent groups and physical activity groups on the study variables.

	**Ds (*N* = 17) *M (SD)***	**CDs (*N* = 21) *M (SD)***	**Cs (*N* = 42) *M (SD)***	***F***	***p***	**η*^2^***	**Tukey HSD^*a*^**	**NPA (*N* = 19) *M (SD*)**	**OPA (*N* = 26) *M* (*SD*)**	**NOPA (*N* = 35) *M* (*SD*)**	***F***	***p***	**η*^2^***	**Tukey HSD^a^**
Psychoticism	5.29 (2.31)	6.57 (1.88)	4.86 (2.14)	3.03*	0.042	0.08	C < CD	5.74 (2.51)	4.5 (1.7)	5.89 (2.23)	2.52	0.087	0.06	OPA < NOPA
Neuroticism	9.47 (2.81)	12.67 (4.29)	10.14 (3.17)	2.86	0.064	0.07	C < CD	10.95 (2.95)	9.19 (2.65)	11.6 (4.22)	1.28	0.284	0.03	OPA < NOPA
School N	15.76 (7.44)	21.38 (9.45)	13.71 (8.97)	1.99	0.144	0.05	D, C < CD	18.79 (9.15)	10.23 (5.9)	19.14 (9.5)	2.03	0.138	0.05	OPA < NPA; NOPA
Rebelliousness	6.06 (2.56)	8.00 (2.32)	5.60 (2.67)	4.89*	0.011	0.18	C < CD	5.53 (2.65)	6.08 (2.57)	6.94 (2.80)	1.00	0.370	0.02	–
Sensorimotor C	1.52 (1.58)	2.76 (2.21)	5.14 (2.31)	15.6*	0.000	0.30	D, CD < C	2.94 (2.12)	5.73 (2.18)	2.71 (2.37)	7.16*	0.001	0.16	NPA, NOPA < OPA

School N, School neuroticism; Sensorimotor C, Sensorimotor coordination; Ds, Disabilities; CDs, Conduct disorder; Cs, Control group; NPA, No physical activity; OPA, Organized physical activity; NOPA, Non-organized physical activity. All the Tukey’s HSD post-hoc are significant at * p < 0.05.

Additionally main effects were obtained in the physical activity group showing that: (a) NOPA reported significantly higher scores of psychoticism and neuroticism than OPA, and (b) OPA reported significantly lower scores of school neuroticism and significant higher scores of sensorimotor coordination than Ds and CDs. The effect size found on physical activity groups was small, η^2^ = 0.16 for sensorimotor coordination (NPA, NOPA < OPA).

Comparing the study variables in interaction of adolescents’ groups × physical activity groups, a significant interaction effect emerged for psychoticism [*F*(2, 73) = 5.05, *p* = 0.009, partial η^2^ = 0.12]. Adolescents with conduct disorder had a higher score compared with Ds and Cs groups. In these results adolescents with conduct disorders were compared only on the NOPA factor. Results showed that for the control group there is no significant difference between groups. In contrast, in the disability group there is a significant difference between NPA and NOPA. Indeed, adolescents from the NPA group had a higher score on psychoticism in comparison with the NOPA group. Another important aspect to consider is the OPA in relation to the variables included in analysis, where adolescents who are involved in OPA had less opportunities to develop maladaptive behaviors during their time at school. All the other variables included in the analysis show the differences between the groups, specifically the OPA. The main effects and interaction effect are presented in Table 3.

**TABLE 3:** Comparison of the study variables across the three groups in interaction with physical activity.

		**Ds M (SD)**	**CDs M (SD)**	**Cs M (SD)**	***F***	***p***	**η*^2^***
Psychoticism	NPA	7.40 (2.07)	–	5.14 (2.44)			
	NOPA	3.71 (1.49)	6.57 (1.88)	6.00 (2.64)	5.05*	0.009	0.12
	OPA	5.40 (1.94)	–	4.29 (1.61)			
Neuroticism	NPA	9.80 (2.38)	–	11.36 (3.10)			
	NOPA	10.43 (3.59)	12.67 (4.29)	9.57 (4.03)	0.645	0.528	0.01
	OPA	7.80 (1.09)	–	9.52 (2.82)			
School N.	NPA	16.80 (7.36)	–	19.50 (9.85)			
	NOPA	15.29 (7.47)	21.38 (9.45)	16.29 (10.64)	1.33	0.270	0.03
	OPA	15.40 (9.04)	–	9.00 (4.35)			
Rebelliousness	NPA	6.60 (2.30)	–	5.14 (2.74)			
	NOPA	4.57 (2.63)	8.00 (2.32)	6.14 (2.91)	2.00	0.142	0.05
	OPA	7.60 (1.81)	–	5.71 (2.63)			
Sensorimotor C.	NPA	1.2 (1.78)	–	3.57 (1.91)			
	NOPA	0.57 (0.78)	2.76 (2.21)	4.71 (2.21)	0.763	0.470	0.02
	OPA	3.2 (0.83)	–	6.33 (1.95)			

Ds, Disabilities; CDs, Conduct disorder; Cs, Control Group; NPA, No physical activity; OPA, Organized physical activity; NOPA, Non-organized physical activity; School N, School Neuroticism; Sensorimotor C, Sensorimotor Coordination. All the Tukey’s HSD post hoc are significant at * p < 0.05.

### Results of Discriminant Function Analyses

**FIGURE 1:**
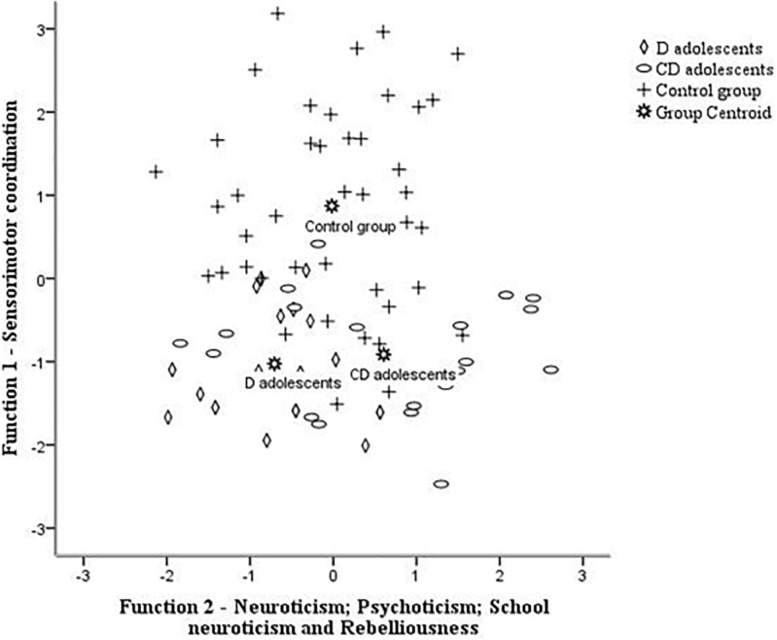
Canonical discriminant function—distribution of adolescents groups based on significant scores of sensorimotor coordination in first function and neuroticism, psychoticism, school neuroticism, and rebelliousness in second function. Ds, disabilities; CDs, conduct disorders; Cs, control group.

For the three groups of adolescents, the discriminant analysis including psychoticism, neuroticism, school neuroticism, rebelliousness and sensorimotor coordination indicated two discriminant functions: F1 (*r*_*can*_ = 50.; Wilks λ = 52.03; *p* < 0.001), explaining 75.1% of the variance, canonical *R*^2^ = 0.38, and F2 (*r_*can*_* = 0.82; Wilks λ = 14.59; *p* = 0.006), explaining 24.9%, canonical *R*^2^ = 0.17. Overall, 66.3% of the cases were correctly classified into the three groups of adolescents—47.1, 47.6, and 83.3% for the Ds, CDs, and Cs, respectively. Sensorimotor coordination loaded highly on the first function (*r* = 0.86), whereas neuroticism (*r* = 0.70), rebelliousness (*r* = 0.57), school neuroticism (*r* = 0.48), and psychoticism (*r* = 0.46) loaded on the second function. As indicated in Figure 1, the discriminant function plot showed that the first function differentiated the Cs group from the CDs and Ds, whereas the second function differentiated the Ds from Cs and Cs from CDs group.

**FIGURE 2:**
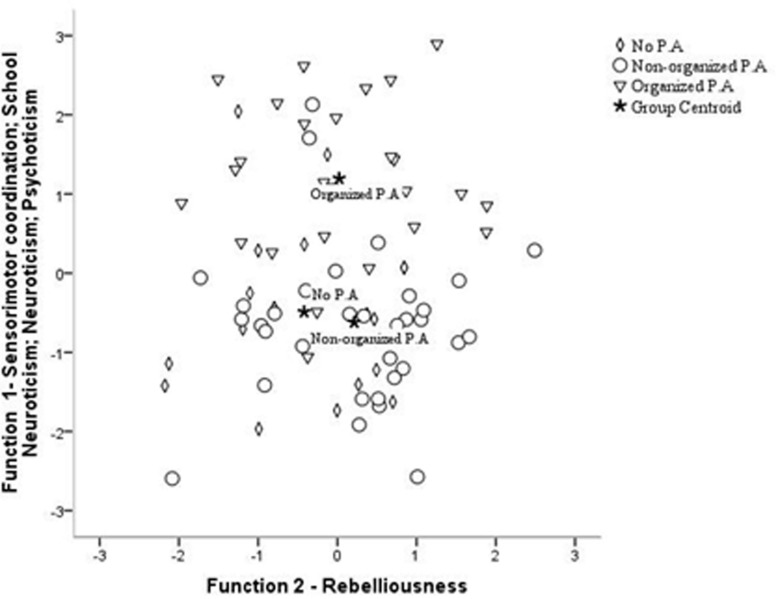
Canonical discriminant function—distribution of physical activity groups based on the significant scores of sensorimotor coordination, school neuroticism, neuroticism, psychoticism, in first function and rebelliousness in the second function. OPA, Organized physical activity; NPA, No physical activity; NOPA, Non-organized physical activity.

For the three groups of physical activity, the discriminant analyses including psychoticism, neuroticism, school neuroticism, rebelliousness and sensorimotor coordination also indicated two discriminant functions: F1 (*r*_*can*_ = 0.56; Wilks λ = 43.64; *p* < 0.001), explaining for 91.9% canonical *R*^2^ = 0.39, and F2 (*r_*can*_* = 0.94; Wilks λ = 4.61; *p* = 0.353) explaining 8.2% of the variance, canonical *R*^2^ = 0.05. Altogether, 65% of the cases were correctly classified into the three groups of physical activity—26.3, 74.3, and 80.8% for the NPA, NOPA, and OPA. Sensorimotor coordination (*r* = 0.75), school neuroticism (*r* = −0.60), neuroticism (*r* = −0.36), and psychoticism (*r* = −0.35) loaded on the first functions, whereas rebelliousness (*r* = 0.84) highly loaded on the second function. As indicated in Figure 2, the discriminant function plot showed that in the first function the OPA group differentiated from the NOPA and NPA, whereas the second function differentiated the NPA from OPA, and OPA from NOPA group.

## Discussion

As mentioned in the literature review, although physical activity generally has been shown to have positive effects on physical and mental health, the influence of organized physical activity on school adaptive behavior of adolescents with disabilities and/or behavioral disorders remains unclear. Therefore the present research investigated the effects of organized physical activity on school behavior, psychological variables (personality factors), and sensorimotor coordination in different groups of adolescents. The three groups consisted of adolescents with disabilities, with conduct disorders, and individuals without diagnosed disabilities or conduct disorders as a control group. Increased knowledge of the role of physical activity in relation to these variables could potentially help teachers foster school integration among adolescents who suffer from Ds and CDs.

### Organized Physical Activity on Personality Traits and School Behavior

The results of the study provided insight into the facilitating role of organized physical activity regarding adolescents’ school adaptability. In particular, adolescents who practiced organized physical activity reported significantly lower scores of psychoticism and neuroticism than their counterparts who were not involved in organized physical activity. Moreover, adolescents who practiced organized physical activity reported significantly lower scores of school neuroticism in comparison to the adolescents who practiced non-organized physical activity or who did not practice physical activity at all. These results are consistent with previous research indicating, that school neuroticism mainly occurs among adolescents who do not practice any physical activity in comparison with those who do practice organized physical activity (Berse et al., 2014). Taken as a whole, our results provided evidence for the positive relationship between organized physical activity and only a reduced level of maladaptive behavior (Carrasco et al., 2006; Rockhill et al., 2009; Esteban-Cornejo et al., 2015). Thus, the benefits of organized physical activity programs among adolescents should be further explored in the future through the prism of the remote effects of decreased recidivism (Rockhill et al., 2009). As expected, results of the present study also showed that adolescents with conduct disorder reported significantly higher levels of school neuroticism, rebelliousness, psychoticism, and neuroticism than adolescents with disabilities and/or the control group. Results also suggested that the risk of school in-adaptability (inferred from psychological and behavioral school outcomes) appeared to be higher among the adolescents with conduct disorders and on the adolescents who do not practice physical activity compared to those who are physically active. Hence, based on the facilitating role of organized physical activity regarding adolescents’ psychological and behavioral school adaptability, it can be concluded that adolescents with conduct disorders could benefit the most from organized physical activity programs.

### Organized Physical Activity on Sensorimotor Coordination

Regarding the sensorimotor coordination of the upper limbs, results of the present study showed a significant advantage for adolescents practicing organized physical activity. In particular, adolescents who practiced organized physical activity had significantly higher scores of sensorimotor coordination in comparison to the adolescents who practiced non-organized physical activity or who did not practice physical activity. However, contrary to our hypothesis, sensorimotor coordination in adolescents with conduct disorders was not significantly different from adolescents with disabilities. Even if the level of coordination seemed to be slightly less developed among Ds participants and CDs participants, adolescents who did not practice physical activity showed even lower scores of sensorimotor coordination. This result not only demonstrated the benefits of practicing physical activity for motor skills, as suggested in other studies (Dodge and Lambert, 2009; Walters et al., 2009; Mangerud et al., 2014; Mosoi and Balint, 2015), but also highlighted that not practicing physical activity may restrict the level of sensorimotor coordination in adolescents period (Quatman-Yates et al., 2012). These results are once again consistent with the risks for adolescents who do not practice physical activity found in previous studies (Burnett-Zeigler et al., 2012; Eime et al., 2013).

Overall, practicing physical exercise in after school programs with an organized framework should be an interactive way for adolescents to manage their free time. One benefit for taking part in an organized physical activity after school program could be the improvement of friendships between peers, as well as the reduction of risk for aggressive behavior or arrest, especially for adolescents with conduct disorders (Jewell et al., 2015; Simplican et al., 2015). These benefits likely develop the usefulness and responsibility of the individual in the team and in the competitive environment.

### Limitations

To the best of our knowledge, the present study is the first survey on school integration via organized physical activities involving adolescents with disabilities and conduct disorders. Therefore, the generalizability of its results is subject to a number of limitations. For example, the cross–sectional nature of this study does not allow for examination of directionality, therefore it remains unclear whether or not the organized school physical activity caused a change in school behavior. Furthermore, the relatively small number of participants in each of the groups (of adolescents and physical activities) and the use of self-reports could limit the generalizability of the results. In this study, the participants with conduct disorders were limited to unstructured activities due to their hospitalization status, which means the after school organized activities were missing. Therefore, in particular, future studies would need to include participants with conduct disorder who do not participate in any form of physical activity. Hence, applied research should conduct a longitudinal study with larger samples. This could provide deeper insights for understanding the effect of organized physical activities in after school programs on school adjustment, especially among adolescents with disabilities and conduct disorders. Another limitation refers to the cultural context in which the study was conducted (i.e., Romanian schools). Our results should not be interpreted in isolation especially about disadvantaged adolescents groups but rather under consideration of previous studies as a contribution to the growing body of evidence on the importance of participating in organized physical activity at school and in after school program. Additionally, futures studies should attempt to replicate these findings in other countries or different types of schools.

### Implications for School Health

In order to foster adolescents’ adaptability to school, personality traits, school behavior and sensorimotor coordination need to be observed and addressed concurrently. Sessions of organized physical activities could prevent the development of antisocial behavior, as well as the aggravation of the school environment. Involvement of adolescents in an organized physical activity after school program might be a particularly promising prevention strategy. Hence, to promote school adjustment, teachers should include didactic strategies for sport physical activities to increase the involvement of adolescents with conduct disorders, as well as disabled adolescents, in organized physical activities with help from families as well as the community (Edwards, 2015; Simplican et al., 2015). The adaptation and implementation of organized physical activities grounded within school programs represents one of the most important social desiderata in order to attract adolescents who do not practice any physical activity (Butler-Kort and Hagewann, 2011; Jewett et al., 2014; Nair et al., 2015). The development of organized physical activity within educational settings could represent a successful approach to public health in the long run (Abula et al., 2016; Marques et al., 2016) for a better adaptation of adolescents to the school and social environment (Bacarro et al., 2012; Drake et al., 2015; Hills et al., 2015), therefore preventing poor mental and physical health in young adulthood (Jewett et al., 2014).

## Conclusion

In conclusion, the results of the present study point out the importance of being physically active as well as being involved in physical activity programs during adolescent years. Particularly, for behavioral problems of adolescents such as rebelliousness and school neuroticism as well as deficits in motor functions (poor sensorimotor coordination), organized physical activity in after school programs seem to offer potential for ameliorating these problems. Despite some limitations, our results also provide valuable information about the risks of adolescents who do not practice any physical activity. Arguably, decreasing strategies to manage organized physical activity decline may favor negative adaptive psychological and motor coordination outcomes similar to adolescents in Ds group and CDs group. Additional, applied research needs to be conducted, that focuses on personality traits (i.e., psychoticism and neuroticism) and school behavior (i.e., school neuroticism) among adolescents who did not practice physical activity in comparison to Ds and CDs adolescents who practiced physical activity. Future research concerning school behavior and organized physical activity could provide insight into whether participating in physical activities is associated with developing antisocial behavior. Furthermore, additional research could also potentially find a relationship between the reduction of sensorimotor coordination and the amount of free time experienced in adolescence. With this knowledge, adolescents could spend their free time furthering their development of sensorimotor coordination.

## Data Availability Statement

The raw data supporting the conclusions of this article will be made available by the authors, without undue reservation, to any qualified researcher.

## Ethics Statement

This study was conducted in accordance with the Declaration of Helsinki. Written informed consent from all subjects was obtained and all data were rendered completely anonymous. The data of this study are open to the public and made available by the Transilvania University of Braşov through the project POSDRU/159/1.5/S/134378.

## Author Contributions

The original research was part of the post Ph.D. thesis of AAM, supervised by LB. AAM, wrote the first draft of the manuscript. GM, JB, and AM wrote sections of the manuscript. All authors made substantial contributions in conceptualization, methodology, to analysis and interpretation of the data, contributed to manuscript revision, read and approved the submitted version.

## Conflict of Interest

The authors declare that the research was conducted in the absence of any commercial or financial relationships that could be construed as a potential conflict of interest.
