# Corrigendum: Influence of Organized vs Non Organized Physical Activity on School Adaptation Behavior

**DOI:** 10.3389/fpsyg.2020.633197

**Published:** 2020-12-17

**Authors:** Adrian A. Mosoi, Jürgen Beckmann, Arash Mirifar, Guillaume Martinent, Lorand Balint

**Affiliations:** ^1^Department of Psychology, Education and Teacher Training, Faculty of Psychology and Education Sciences, Transilvania University of Braşov, Braşov, Romania; ^2^Department of Sport and Health Sciences, Chair of Sport Psychology, Technical University of Munich, Munich, Germany; ^3^Faculty of Health and Behavioural Sciences, School of Human Movement and Nutrition Sciences, University of Queensland, Brisbane, QLD, Australia; ^4^Department of Physical Education and Sport Sciences (PESS), University of Limerick, Limerick, Ireland; ^5^Laboratory of Vulnerabilities and Innovation in Sport, University of Claude Bernard Lyon 1 – University of Lyon, Lyon, France; ^6^Department of Physical Education and Special Motricity, Faculty of Physical Education and Mountain Sports, Transilvania University of Braşov, Braşov, Romania

**Keywords:** physical activity, disabilities, conduct disorders, school behavior, adolescents

In the published article, the order of the first name and the surname of the authors was incorrect. The first name of the first author was swapped with his middle name.

The names of the authors were incorrectly spelled as **Mosoi A. Alexandru, Beckmann Jürgen, Mirifar Arash, Martinent Guillaume and Balint Lorand**.

The correct spelling of the authors' names is **Adrian A. Mosoi, Jürgen Beckmann, Arash Mirifar, Guillaume Martinent and Lorand Balint**.

The authors apologize for this error and state that this does not change the scientific conclusions of the article in any way. The original article has been updated.

